# Supplementary material for: Analysis of PPARγ Signaling Activity in Psoriasis
Source: Int J Mol Sci. 2021 Aug 10;22(16):8603. doi: 10.3390/ijms22168603 (PMC8395241; doi:10.3390/ijms22168603)
Supplement: Supplementary file 1 [file ijms-22-08603-s001.zip › Supplemental materials_Analysis of PPARg signaling activity in psoriasis/Pathway models/Models images and html files/Differentiation of psoriatic T cells/189827.html]

Th2 immune response


# Cell Process Th2 immune response

|  |  |
| --- | --- |
| URN | urn:agi-gocellproc:0042092 |
| Total Entities | 0 |
| Connectivity | 368 |
| Name | Th2 immune response |

---

|  |  |
| --- | --- |
| Pathway | Basophil Activation |
|  | Atopic Dermatitis Onset |
|  | Mast-Cells Activation in Atopic Dermatitis |
|  | T-Cells Differentiation Block in Psoriasis |
|  | Basophil Activation in Asthma |
|  | Th17-Cell and Th1 Immune Responsein Psoriatic Arthritis |
|  | PPAR Psoriasis |
|  | 1\_Differentiation of psoriatic T cells |
|  | 1\_The onset of atopic dermatitis |
|  | Differentiation of psoriatic T cells |

---

|  |  |
| --- | --- |
| MedScan ID | 4142092 |

---

|  |  |
| --- | --- |
| Alias | Th2 immune response |

---

|  |  |
| --- | --- |
| GO ID | 0042092 |

---
